# The Effect of β-Glucan on the Release and Antiradical Activity of Phenolic Compounds from Apples in Simulated Digestion

**DOI:** 10.3390/molecules30020301

**Published:** 2025-01-14

**Authors:** Lidija Jakobek, Daniela Kenjerić, Lidija Šoher, Petra Matić

**Affiliations:** Faculty of Food Technology Osijek, Josip Juraj Strossmayer University of Osijek, Franje Kuhaca 18, 31000 Osijek, Croatia; daniela.kenjeric@ptfos.hr (D.K.); lidija.soher@ptfos.hr (L.Š.); petra.matic@ptfos.hr (P.M.)

**Keywords:** gastric digestion, intestinal digestion, *Malus domestica*, dietary fiber, food matrix, adsorption, HPLC

## Abstract

Beneficial activities of phenolic compounds in the gastrointestinal tract, such as antiradical activity, are affected by the food matrix. The aim of this study was to investigate the influence of one constituent of the food matrix (dietary fiber β-glucan) on the release and antiradical activity of phenolic compounds from apples in gastrointestinal digestion. Simulated digestion in vitro was conducted on whole apples without or with added β-glucan. Antiradical activity was determined with the DPPH method. The total amount of released phenolic compounds in the stomach (563 mg kg^−1^ fresh weight (fw), 85%) decreased in the intestine (314 mg kg^−1^ fw, 47%) (*p* < 0.05). The presence of β-glucan decreased the release of phenolic compounds to 80 and 74% in the stomach and to 44 and 40% in the small intestine when there were lower and higher β-glucan amounts, respectively. A statistical analysis showed differences between release in digestion without or with β-glucan. B-glucan adsorbed up to 24 (stomach) and 32 mg g^−1^ (small intestine) of the phenolics. Phenolic compounds scavenged more free radicals in the small intestine than in the stomach, and β-glucan decreased this activity, but not significantly. The interaction between β-glucan and phenolic compounds should be considered when explaining the beneficial effects in the stomach and small intestine.

## 1. Introduction

The beneficial effects of phenolic compounds on the human body have been studied for decades. The first system that comes into contact with phenolic compounds is the gastrointestinal tract. Due to relatively high amounts of phenolic compounds in all parts of the gastrointestinal tract, this system is a logical place for their potential beneficial effects to be realized. Many studies have shown the beneficial effects of phenolic compounds on the gastrointestinal tract [1,2,3,4,5,6,7,8,9,10]. Gastric cancer, an aggressive disease that causes a high number of deaths globally [1], is a condition in which phenolic compounds can be helpful [1]. It has been shown that they depress the growth of stomach cancer cells in vitro [2] and reduce the proliferation of gastric cancer [1]. Moreover, phenolic compounds attenuate *Helicobacter pylori* infection [3,4], a risk factor for gastric cancer [4]. Another stomach condition that causes health problems is gastric ulcer, a condition of an injured gastric mucosa. Phenolic compounds from propolis [5] or ellagic acid [6] showed the possibility to heal gastric ulcers by stimulating mucus production and by improving antioxidant status [5]. The potential for beneficial effects in the small intestine is large. Phenolic compounds might help in maintaining proper intestinal barrier function [7,8]. They can inhibit enzymes important for carbohydrate metabolism, such as α-amylase and α-glucosidase [9,10], which can lead to lower blood glucose levels. Apples are fruits with a high frequency of consumption in the human diet and are available year-round, so their phenolic compounds are available and are helpful in improving the health of the gastrointestinal tract. Phenolic compounds specifically from apples have shown the potential for many beneficial effects, as explained in a review paper [11]. They can alleviate changes caused by nonsteroidal anti-inflammatory drugs, help achieve gastric ulcer healing, or alleviate problems in the stomach connected with *Helicobacter pylori* [11]. In the small intestine, phenolic compounds from apples can alleviate changes caused by nonsteroidal anti-inflammatory drugs, have beneficial effects on hyperglycemia, and they can strengthen the gut barrier [11].

Many of the mentioned diseases arise from an imbalance between reactive oxygen species and the antioxidant defense system [12]. In cases of excessive use of nonsteroidal anti-inflammatory drugs, smoking, alcohol, or *H. pylori* infection, epithelial cells in the stomach can generate reactive oxygen species to fight against those factors. However, reactive species can target host cells, too. An antioxidant system is an endogenous defense system against reactive oxygen species. If an antioxidant system is weakened, then higher amounts of reactive species can cause oxidative stress, which can result in gastric inflammation, ulcers, or cancer [12]. Oxidative stress can cause some problems in the small intestine, too. In the small intestine, gluten peptides can induce oxidative stress that can have cytotoxic effects on epithelial cells (celiac disease). In inflammatory bowel disease, oxidative damage is detected in the intestinal mucosa [12]. Polyphenols can oxidize [12,13], and with this activity, they can neutralize reactive species, which makes them helpful in the fight against the oxidative stress caused by those reactive species. Many earlier works have studied the antioxidant role of polyphenols [14,15,16,17].

The mentioned activities of phenolic compounds can be affected by the presence of the food matrix. Dietary fibers are constituents of the food matrix that stay undigested in the stomach and small intestine and eventually ferment in the colon. Due to their presence in the stomach and small intestine, they have the possibility to interact with phenolic compounds and affect their activities. In particular, β-glucans are fibers found in cereals, such as barley or oats, and in other sources (such as yeasts or mushrooms). The basic unit in β-glucans is a D-glucose unit, linked with β-glycosidic bonds. They can create gels and contribute to viscosity. These characteristics depend on the molecular weight, the source of β-glucan, or their ability to form aggregates [18]. More specifically, in β-glucans from cereals, glucose units are linked with β-(1–3) and β-(1–4) linkages, macromolecules have a rod-like conformation with a molecular mass ranging from 31 to 3100 × 10^3^ g mol^−1^ [18], and they are soluble in water. Cereals can be used in the diet together with fruits, which are rich sources of phenolic compounds. In other words, β-glucans and phenolic compounds can be found together in the gastrointestinal tract and interact, which can affect the beneficial activities of both of these groups of compounds. However, to the best of our knowledge, the effects of β-glucans on the antioxidant activity of phenolic compounds from apples have not been studied.

Our aim is to study the effect of β-glucan on the antiradical activity of phenolic compounds from apples released in the stomach and small intestine in vitro. The gastrointestinal digestion of plain apples (as a control) and apples with different concentrations of β-glucan was conducted in vitro by simulating the digestion process in the stomach and small intestine. The antiradical activity of phenolic compounds released in the stomach and small intestine without (control) or with added β-glucan was studied with the DPPH method.

## 2. Results

### 2.1. Phenolic Compounds During Simulated Gastrointestinal Digestion

Table 1 shows the amounts of phenolic compounds present in apples before digestion. The apples contained anthocyanins (32 mg kg^−1^ fw), flavan-3-ols (367 mg kg^−1^ fw), dihydrochalcones (40 mg kg^−1^ fw), phenolic acids (139 mg kg^−1^ fw), and flavonols (84 mg kg^−1^ fw). These are phenolic groups usually found in apples, and the amounts are in accordance with the data in the literature [19,20]. The amounts of total phenolic compounds in native apples (662 mg kg^−1^ fw) and those released at the end of gastric digestion (563 mg kg^−1^ fw) were similar. However, they decreased toward the end of the small intestine (314 mg kg^−1^ fw) (*p* < 0.05). This decrease is in accordance with previous studies [16,21,22]. The amounts of some individual phenolic groups decreased in the stomach (anthocyanins, dihydrochalcones, and flavonols; *p* < 0.05), and the amounts of most of them additionally decreased in the small intestine (anthocyanins, flavan-3-ols, dihydrochalcones, and flavonols; *p* < 0.05) in comparison to the amounts in native apples. Furthermore, in a comparison of the gastric and small intestinal phases, it can be seen that the amounts of total phenolic compounds significantly decreased (*p* < 0.05) from the gastric phase to the end of the small intestinal phase. The amounts of most of the phenolic groups found in the stomach and small intestine were similar, while the amounts of flavan-3-ols decreased significantly from the stomach to the small intestine (*p* < 0.05).

There were some differences in the individual phenolic compounds between the two phases of digestion (Table 1). Cyanidin-3-glucoside was not identified in the small intestinal phase, similar to earlier studies [21,22]. However, it was present in the gastric phase. At a lower pH, such as in the gastric phase (pH 3), anthocyanins are present in the form of flavylium cation, which is the reason for their stability in the stomach. At a higher pH, such as in the small intestinal phase (pH 7), they transform into a carbinol pseudo-base form, which is colorless [23]. This leads to their presence in a lower detected amount or disappearance in the small intestine. There were some differences in the flavan-3-ol group. Procyanidin B1, which was identified in the stomach, could not be identified in the small intestine, while the amount of procyanidin B2 was lower (*p* < 0.05). Different behaviors can be the result of different pK_a_ values. When the pH is the same value as the pK_a_, 50% of a compound is in a dissociated form, and 50% is in a non-dissociated form. At a pH lower than the pK_a_, the compound is mostly in a non-dissociated form, while at a pH higher than the pK_a_ value, the compound is mostly in dissociated forms. The pK_a_ values of some flavan-3-ols (catechin pK_a_ values of 8.68, 9.70, and 11.55; epicatechin values of 8.91, 9.93, and 11.76) [24] suggest that they might be present in non-dissociated forms in the stomach (pH 3) and small intestine (pH 7), and they should be identified and quantified. A possible reason for their lower amount in the small intestine might not be their dissociation but rather their potential degradation into unknown products, as suggested in an earlier study [16]. Two new phenolic acids not present in gastric digestion were identified in the small intestine, neochlorogenic acid and cryptochlorogenic acid. These phenolic acids are the result of the isomerization of chlorogenic acid at the higher pH in the small intestine, similar to what was found in earlier studies [25]. Moreover, it was found that at pH 5–5.5, chlorogenic acid isomerizes into cryptochlorogenic acid, and at pH 6–9, it isomerizes into cryptochlorogenic and neochlorogenic acid [26]. These findings agree with the results of this study and confirm the isomerization of chlorogenic acid into neochlorogenic and cryptochlorogenic acid in the small intestine at pH 7. Of the 110 mg kg^−1^ of chlorogenic acid found in gastric digestion, 97.7 mg kg^−1^ was recovered in the small intestine as chlorogenic, neochlorogenic, and cryptochlorogenic acids, which is 88.8% of the amount found in gastric digestion. This suggests the degradation of chlorogenic acid of around 11% in the small intestine. Flavonols and dihydrochalcones showed stability in the small intestine, similar to earlier studies [25]. The pK_a_ values of some flavonols (quercetin in aqueous medium; pK_a_ 1.8, 6.4, 8.1, 9, 9.6, 11.3) [27] indicate that they might be found in multiple forms at different pH values. However, at the pH value of the stomach (pH 3), they might be present in non-dissociated forms [27], and at the pH of the small intestine (pH 7), the dominant form is the form that has one dissociated OH group (3 position) [27]. Regardless of the possible dissociation of one OH group, flavonols were detected at pH 3 and 7 and did not show a decrease in their amount, passing from the stomach to the small intestine.

**Figure 1:**
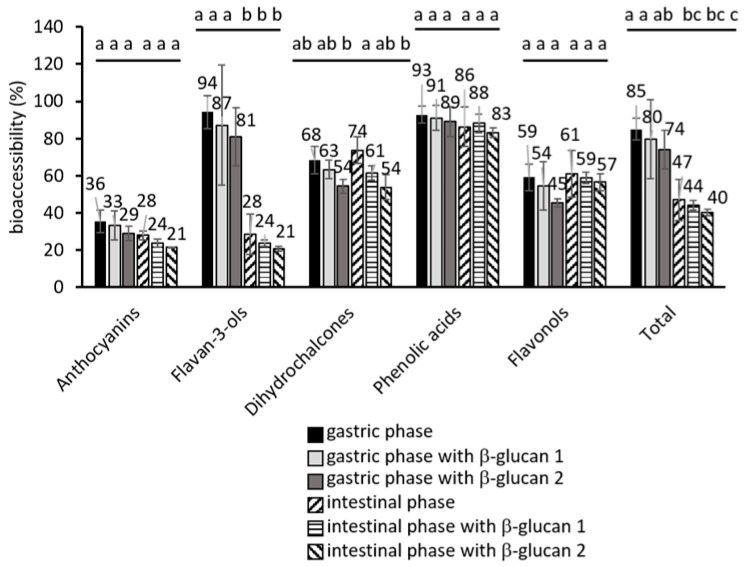
Bioaccessibility of phenolic groups in gastric and intestinal digestion, expressed as recovery (%). Different lowercase letters above each phenolic group represent a significant difference (*p* < 0.05) obtained with the post-hoc Tukey test.

Phenolic compounds were available for absorption in the stomach (Figure 1). The bioaccessibility of total phenolic compounds reached 85% in the stomach and decreased significantly in the small intestine to 47% (*p* < 0.05).

Similar results were reported in earlier studies [25]. At least 15% of phenolic compounds were not liberated from the food matrix in the stomach. It can be suggested that those phenolic compounds have the possibility to be liberated in the small intestine; however, they the possibility to be degraded due to the conditions of elevated pH in the small intestine. It can also be suggested that these phenolic compounds have the ability to be carried to the colon, where they can show potential beneficial effects.

Figure S1 shows the percentage distribution of phenolic groups in apples before digestion and during gastrointestinal digestion. The composition in the stomach shows similarities with the composition of native apples, with the exception of lower percentages of anthocyanins and flavonols (*p* < 0.05). Dominating compounds in the stomach were flavan-3-ols and phenolic acids. The composition in the small intestine changes with dominating flavan-3-ols, phenolic acids, and flavonols. Similarly, in an earlier study, after the digestion of apples, flavonols and hydroxycinnamic acids were the major compounds [14].

The overall results suggest a significant decrease in the released phenolic compounds from the stomach to the small intestine. The conditions in the small intestine encourage some changes in individual compounds. Some flavan-3-ols might degrade, while chlorogenic acid isomerizes into cryptochlorogenic acid and neochlorogenic acid. Anthocyanins change their form at higher pH values of the small intestine. Flavonols and dihydrochalcones show stability in the small intestine. It can be suggested that the main reason for the lower total amounts of phenolic compounds in the small intestine is the degradation of flavan-3-ols due to their significant decrease from the gastric phase to the end of the small intestinal phase.

### 2.2. The Influence of β-Glucan on the Released Phenolic Compounds

**Figure 2:**
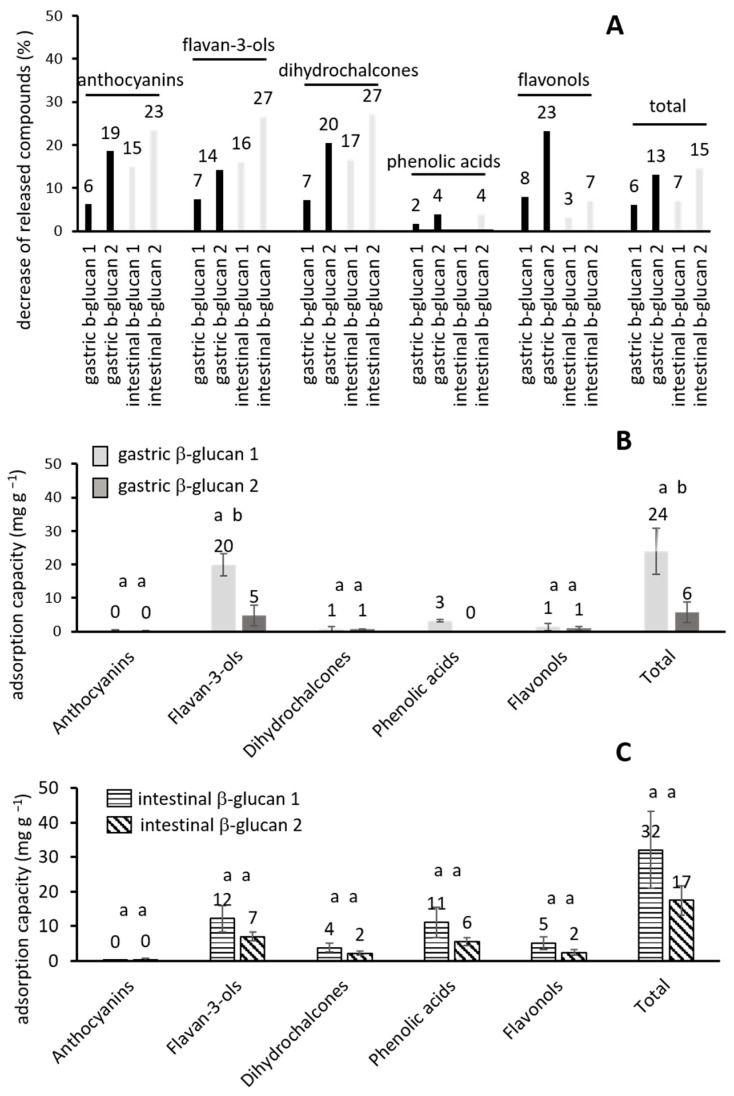
(**A**) The decrease in released phenolic groups in gastric and intestinal digestion with added β-glucan in comparison to the amount released without the addition of β-glucan; (**B**) the adsorption capacity (mg g^−1^ β-glucan) in gastric and (**C**) intestinal digestion. Different lowercase letters above each column represent a significant difference (*p* < 0.05) obtained with the post-hoc Tukey test.

The amounts of released phenolic compounds in digestion with β-glucan added in two different concentrations are shown in Table 1. In the stomach and in the small intestine, the addition of β-glucan at both concentration levels did not significantly influence the release of phenolic groups (Table 1) or bioaccessibility (Figure 1). However, a small decrease in the release of phenolic compounds was noticed, and this decrease is shown in Figure 2A (2 to 23% in the stomach, 3 to 27% in the small intestine). The decrease could be the result of the adsorption of phenolic compounds onto β-glucan. A previous study showed that various phenolic compounds from apples have the possibility to adsorb onto β-glucan [21]. Similarly, earlier studies showed the adsorption of tea polyphenols onto β-glucan [28,29].

Figure 2B,C show the adsorption capacities of β-glucan in the digestion process. When a lower amount of β-glucan was added in gastric digestion (β-glucan 1) (Figure 2B), the amounts of all adsorbed phenolic groups and total polyphenols reached a total adsorbed amount of 24 mg per g of β-glucan. The addition of a higher amount of β-glucan (β-glucan 2) decreased the adsorption capacities, which was statistically significant for flavan-3-ols and total adsorbed phenolics (*p* < 0.05). The total adsorbed amount reached 6 mg g^−1^ β-glucan. It can be suggested that the increase in the amount of β-glucan leads to the decrease in its adsorption capacity. When a higher concentration of β-glucan is present in digestion, the adsorbed phenolic compounds are distributed over this higher amount, which results in a lower adsorbed amount per mass of β-glucan (lower adsorption capacity). Similar results can be seen in the small intestine (Figure 2C). When a lower amount of β-glucan was added (β-glucan 1), the adsorbed amounts reached 32 mg per g of β-glucan. With a higher amount of added β-glucan (β-glucan 2), the total adsorbed amount decreased and reached 17 mg per g β-glucan. Earlier studies suggested the creation of H bonds and Van der Waals forces between phenolic compounds and β-glucan [28,29]. Those bonds can be suggested for this study, between OH groups of β-glucan and OH groups of phenolic compounds.

### 2.3. A Statistical Analysis of the Results

To more clearly show the difference between the released phenolic compounds in different phases of digestion and the influence of β-glucan on release and bioaccessibility (recovery), the results were analyzed with a regression analysis and a principal component analysis (PCA). First, regression lines were constructed as the amounts of phenolic compounds released during digestion vs. all phases of digestion without or with added β-glucan. An example of a regression line is shown in Figure S2, and the results are shown in Table S1. The regression lines of the phenolic groups and total phenolics released through all phases of digestion had negative slopes (Table S1). The highest values of negative slopes were found for the total phenolic compounds (−69.60) and flavan-3-ols (−57.26). All negative slopes are statistically significant (*p* < 0.05) and indicate a statistically significant decrease in phenolic compounds during the digestion phases. After that, a regression analysis was conducted for the released phenolic compounds vs. the different phases of digestion and the presence of β-glucan (Table S2). A significant effect of β-glucan on the decrease in phenolic compounds was shown for some phenolic groups (anthocyanins and dihydrochalcones, *p* < 0.05). Finally, to further study the influence of β-glucan, a sign test was conducted on the drop of phenolic compounds in gastric and intestinal digestion caused by adding β-glucan (Table S3). These drops in amounts reported in Table S3 were obtained by subtracting the mean amounts of released phenolic groups without β-glucan and those of released phenolic groups when β-glucan was added to digestion. Of the 20 observations carried out, 19 were negative, indicating that the amount of released phenolic groups was decreased by adding β-glucan. The sign test *p* value was 0.00002 (arising from the binomial distribution probability of at least 19 out of 20 negative signs if the null hypothesis of equi-probable signs were true). This indicates a strong influence of β-glucan on the decrease in the release of phenolic compounds in the gastric and small intestinal phases of digestion.

**Figure 3:**
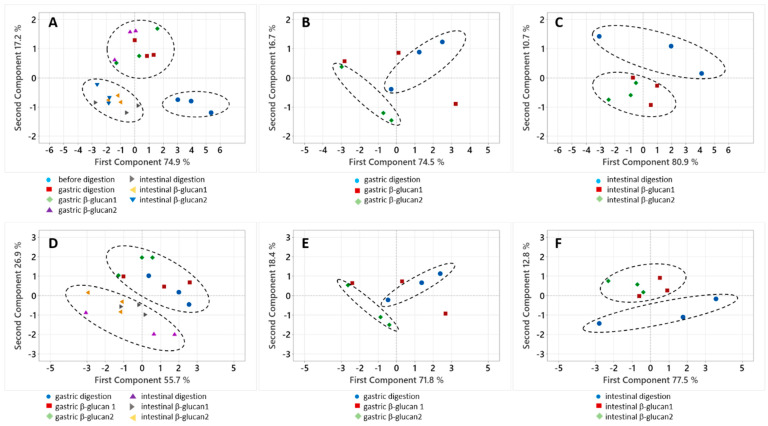
A principal component analysis of the amounts of total anthocyanins, flavan-3-ols, dihydrochalcones, phenolic acids, and flavonols (**A**) before digestion and in all phases of gastric and intestinal digestion, (**B**) in gastric digestion, and (**C**) in intestinal digestion. A principal component analysis of the bioaccessibility of total anthocyanins, flavan-3-ols, dihydrochalcones, phenolic acids, and flavonols (**D**) in all phases of gastric and intestinal digestion, (**E**) in gastric digestion, and (**F**) in intestinal digestion. Dotted lines surround observed grouping of data.

The amounts of phenolic compounds in apples and released in digestion are shown in Figure 3A–C, which were created by graphing onto the first two principle components in the principle component analysis (PCA). Clear grouping of the amounts of phenolic compounds in apples and those released in the stomach and small intestine can be seen (Figure 3A). When only the amounts released in gastric digestion were analyzed with a PCA, the groupings of compounds released without β-glucan and with added β-glucan are not so clear (Figure 3B). Grouping was observed for the amounts released in the small intestine without or with added β-glucan (Figure 3C). The results of the PCA for bioaccessibility are shown in Figure 3D–F, and the results are similar. The data for bioaccessibility were grouped according to bioaccessibility in the stomach and small intestine (Figure 3D). According to the gastric digestion simulation conducted without or with added β-glucan, the grouping was not so clear (Figure 3E). Finally, grouping according to the small intestine digestion simulation conducted without or with added β-glucan was observed (Figure 3F).

The results of regression and PCA suggest that there is a difference between gastric and intestinal digestion in the release and bioaccessibility of phenolic compounds. There are also differences in the release of phenolic compounds between digestion without and with added β-glucan.

### 2.4. Antiradical Activity

The inhibition of DPPH radicals in time is presented in Figure S3. Phenolic compounds from apples and those released in the small intestine showed faster inhibition of DPPH radicals, reaching a steady state after 5 to 10 min. However, those from the gastric phase reacted slower and did not reach a steady state. Since phenolic compounds from apples and those released in the small intestine reached a steady state after up to 10 min, we used the percent inhibition after 10 min to calculate the EC_50_ values, which represent the amount of phenols needed to inhibit DPPH radicals for 50% (Table S4). According to the EC_50_ values, the stronger scavengers of DPPH free radicals were the phenolic compounds from apples (before digestion) and those released in the small intestine in comparison to the phenolic compounds released in the stomach (*p* < 0.05).

In addition, the percent inhibition of free radicals caused by the same amounts of phenolic compounds (0.002 mg) after 5, 10, and 20 min of reaction was calculated, and the results are shown in Table 2. Phenolic compounds from apples and those released in the stomach inhibited free radicals in a similar percentage (14 to 24% of apple phenolics were released before digestion; 12 to 20% were released in the stomach), while those released in the small intestine inhibited free radicals in a higher percentage (28 to 36%). Here, where the ability of the same amount of phenolic compounds to inhibit free radicals was calculated, it can be seen that phenolic compounds in the stomach have a similar ability to neutralize free radicals as phenolic compounds in the apple extract. However, the ability to neutralize free radicals was higher for phenolic compounds in the small intestine. The presence of β-glucan in gastrointestinal digestion lowered the percentage of inhibition, but not significantly.

## 3. Discussion

### 3.1. Phenolic Compounds in Native Apples and in Gastric and Intestinal Digestion

Many potentially positive bioactivities of phenolic compounds from apples were shown in the gastrointestinal tract. In the stomach, phenolic compounds from apples can be helpful in scavenging harmful species generated in the lipid peroxidation process [30], in healing gastric ulcers [31,32], or in alleviating problems caused by *Helicobacter pylori* [33]. Phenolic compounds from apples are released and are bioaccessible in the stomach (Table 1). All phenolic compounds from apples, anthocyanins, flavan-3-ols, dihydrochalcones, phenolic acids, and flavonols can be found in the stomach during digestion but in a somewhat lower amount (563 mg kg^−1^ fw) than naturally present in apples (662 mg kg^−1^ fw) (Table 1). Still, in total, 85% of phenolic compounds were available for absorption or to show bioactivities in the stomach (Figure 1). This gives them a high potential to be bioactive in the stomach. The percentage distribution of phenolic compounds in the stomach is similar to the one in apples, with flavan-3-ols and phenolic acids being the major groups (Figure S1). It might be suggested that flavan-3-ols and phenolic acids might be more important regarding the beneficial effects of apples in the stomach due to their higher amounts.

In the small intestine, some of the effects include alleviating changes caused by nonsteroidal anti-inflammatory drugs and strengthening the gut barrier [8,34]. In addition, phenolic compounds from apples can inhibit the activity of enzymes such as α-amylase or α-glucosidase, which can be helpful in alleviating hyperglycemia [35,36]. Phenolic compounds from apples are present in the small intestine, but in a lower amount (314 mg kg^−1^ fw) than that found in natural apples (662 mg kg^−1^ fw) or in the stomach (563 mg kg^−1^ fw) (Table 1). Due to a higher pH, some phenolic compounds biotransform in the small intestine, like anthocyanins, which change their structure at a higher pH, or phenolic acids, which isomerize into other forms (chlorogenic acid into neochlorogenic and cryptochlorogenic acids). Some phenolic compounds degrade into unknown products like flavan-3-ols. Other groups showed better stability, such as flavonols and dihydrochalcones. However, in total, 47% of phenolic compounds were available for absorption in the small intestine (Figure 1), which is still a high percentage. This gives them the potential to be bioactive in the small intestine. The percentage distribution of phenolic compounds also changed in the small intestine. The dominant groups were phenolic acids, flavan-3-ols, and flavonols (Figure S1). When considering the beneficial effects of apples on the small intestine, flavan-3-ols, flavonols, and phenolic acids might be more important.

### 3.2. Gastrointestinal Digestion with or Without β-Glucan

Dietary fibers, as one of the constituents of the food matrix, have the potential to affect the release of phenolic compounds and their bioactivities. To study the influence of dietary fiber, β-glucan from barley, a soluble β-glucan, was used in this study. Soluble dietary fibers are viscous and ferment in the colon by gut bacteria. We used two different concentrations of β-glucan, a lower one (β-glucan 1) and a higher one (β-glucan 2). This study shows that the addition of β-glucan decreased the amount of total released phenolic compounds in the stomach to 529 or 490 mg kg^−1^ fw depending on whether a lower or higher amount of β-glucan was added, respectively (Table 1). In the small intestine, the results were similar. The addition of β-glucan decreased the amount of total released phenolic compounds to 292 or 268 mg kg^−1^ depending on whether lower or higher amounts of β-glucan were added (Table 1). It can be suggested that β-glucan adsorbed some of the liberated phenolic compounds, which led to lower amounts of released compounds.

Apples contain other naturally present dietary fibers. The total amount of fibers in apples, according to various sources, is around 3% [19,20]. The presence of soluble dietary fibers in apples is up to 0.5 [19] or 1.6% [20]. The rest are insoluble fibers, such as cellulose. The soluble fibers found in apples are pectins, which are present in around 16% in the dry matter of whole apples [37]. Accordingly, pectins were present in the simulated digestion of apples in this study, but in a low amount. In gastric digestion, dry apple material and gastric fluids were present in a ratio of 1:9.4 and in the small intestine in a ratio of 1:21.9. So, the amounts of fibers such as pectin (16% of the dry material) and cellulose (up to 0.6% of the dry material [38]) were small. Pectins might have had a small influence on the amounts of released phenolic compounds. However, since pectin was present in all digestion processes conducted without or with added β-glucan, the effect of β-glucan alone, as seen in this study, could be attributed to β-glucan itself. Furthermore, digestive enzymes are present in digestion. Phenolic compounds have the ability to interact with proteins. However, since enzymes were present in both types of experiments without or with the addition of β-glucan, the decrease in the amount of released phenolics could be attributed to β-glucan itself.

There can be several consequences of the adsorption of phenolic compounds onto dietary fibers during digestion. One consequence can be that dietary fibers can be studied as carriers of phenolic compounds into the colon, where they can be fermented, and phenolic compounds can be liberated, showing potential beneficial effects on the colon. It can be highlighted that including more dietary fibers in the everyday diet can naturally help carry higher amounts of phenolic compounds to the colon. This fact can be helpful for promoting healthy dietary habits. The second consequence might be a beneficial effect of the complexes of dietary fiber–phenolic compounds inside the stomach and small intestine. These complexes need to be further studied. B-glucan itself has shown beneficial effects on the gastrointestinal tract [18,39]. In a study conducted on rats, it was shown that β-glucan can alter the gut microbiota composition, strengthen the epithelial barrier, and promote an increase in beneficial bacteria. However, the effects were dose dependent [39]. The effect of β-glucan should be further studied. It can be suggested that one aspect that merits further study is the interactions of β-glucan with phenolic compounds and its influence on the beneficial effects of β-glucan. Phenolic compounds can be present in the gastrointestinal tract together with β-glucan, which gives them the opportunity to interact with β-glucan.

The intake of total dietary fiber varies among people. According to Stephen et al. (2017) [40], in various countries around the world, the intake of dietary fiber in g/day is 13.6 to 24 in adults, 14.3–21.1 in older adults, 11.6 to 27.1 in children aged 13 to 18 years, 9.4 to 22.9 in children aged 4 to 12 years, and 8.2 to 16.1 in children aged 0 to 4 years. In most of these countries, the principal food sources of fibers are grain products (31.9–49%). The sources of β-glucan specifically are breads made out of whole flour, breakfast cereals and cereal bars, oat porridge, and rye-based products. Due to the intake of fibers in the diet, specifically β-glucans, their interactions with phenolic compounds should be considered.

### 3.3. Antiradical Activity of Phenolic Compounds Released in the Simulated Digestion

Phenolic compounds from apples and those released in the stomach and small intestine showed the ability to scavenge free radicals. The ability to scavenge free radicals was stronger for phenolic compounds released in the small intestine in comparison to the compounds released in the stomach. Earlier studies have obtained somewhat similar results. Similarly to our study, the antiradical activity of apple polyphenols increased from gastric to intestinal digestion [15]. The antiradical activity of apple extract determined with the ABTS method was higher than that of compounds released in gastric digestion and then increased after small intestinal digestion [41]. This result is also similar to that obtained in our study. Opposite to these results, when the antiradical activity was determined after the in vitro digestion of apples with the DPPH method [14,16], antiradical activity decreased after digestion. Phenolic compounds released in digestion with added β-glucan also showed the possibility to scavenge free DPPH radicals. This is similar to an earlier study that showed that phenolic compounds from tea and β-glucan, in a mixture or in complexes, can scavenge free radicals [29].

It can be suggested that different compositions of compounds in the small intestine, where phenolic acids, flavan-3-ols, and flavonols dominated, caused a stronger ability to scavenge free radicals. Pectin, present in apple, probably does not influence the antiradical activity of phenolic compounds after digestion, according to an earlier study [14]. The addition of β-glucan lowered, to some extent, the antiradical activity of phenolic compounds (Table 2), but not significantly. Due to its viscosity, β-glucan might have prevented phenolic compounds from reaching free radicals, which resulted in a lower percentage of DPPH inhibition.

## 4. Materials and Methods

### 4.1. Chemicals and the Preparation of Solutions

The company Gram mol (Zagreb, Croatia) provided potassium chloride, potassium dihydrogen phosphate, sodium hydrogen carbonate, calcium chloride, and magnesium chloride. Ammonium carbonate was purchased from Kemika (Zagreb, Croatia), and sodium chloride from Carlo Erba Reagents (Val de Reuil, France). HPLC-grade solutions of orto-phosphoric acid (85%) and methanol were purchased from Fluka (Buchs, Switzerland) and J.T. Baker (Gliwice, Poland), respectively. Enzymes (α-amylase (A3176, 13 U/mg), pepsin (P7000, 632 U/mg), and pancreatin (P7545, 8 USP)) and bile salt (B 8756, microbiology grade) were purchased from Sigma-Aldrich (St. Louis, MO, USA), as well as phenolic compound standards (quercetin-3-rutinoside; quercetin-3-glucoside; (+)-catechin hydrate; (−)-epicatechin; and chlorogenic, neochlorogenic, cryptochlorogenic, and *p*-coumaric acids), β-glucan from barley, and 2,2-diphenyl-1-picryl-hydrazil (DPPH). Other phenolic compounds were purchased from Extrasynthese (Genay, France) (quercetin-3-galactoside, quercetin-3-rhamnoside, procyanidin B1 and B2, phloretin-2′-*O*-glucoside, cyanidin-3-galactoside chloride, and cyanidin-3-glucoside chloride).

Stock solutions were prepared ((KCl, KH_2_PO_4_, and (NH_4_)_2_CO_3_, 0.5 M), (NaHCO_3_, 1 M), (MgCl_2_, 0.15 M), (NaCl, 2 M), and (CaCl_2_, 0.3 M)) and used for the preparation of simulated salivary fluid electrolyte solution (SSF), simulated gastric fluid electrolyte solution (SGF), and simulated intestinal fluid electrolyte solution (SIF). The final concentrations in mmol L^−1^ were as follows: SSF (18.875 KCl, 4.625 KH_2_PO_4_, 17 NaHCO_3_, 0.056 MgCl_2_, and 0.06 (NH_4_)_2_CO_3_); SGF (8.625 KCl, 1.125 KH_2_PO_4_, 31.25 NaHCO_3_, 0.15 MgCl_2_, 0.625 (NH_4_)_2_CO_3_, and 59 NaCl, and pH was adjusted to 3 with 1 M HCl), and SIF (8.5 KCl, 1 KH_2_PO_4_, 106.25 NaHCO_3_, 0.4125 MgCl_2_, and 48 NaCl, and pH was adjusted to 7 with 1 M HCl). Simulated fluids with electrolytes usually present in digestion were used for simulated gastric digestion and small intestinal digestion of apples according to [42]. Enzymes were prepared each day and preincubated at 37 °C before use (1000 mg L^−1^ α-amylase in SSF, 31,660.61 mg L^−1^ pepsin in SGF, 8000 mg L^−1^ pancreatin in SIF). Bile salts were prepared in SIF (25,000 mg L^−1^).

### 4.2. Apple Samples

‘Red Delicious’ apples were purchased from a local supermarket. Whole apples with peel were cut, the core was removed, and sliced apples were dried in a food dehydrator at a room temperature using a flow of air (KYS-328A, Delimano, Guandong Kangye Electric Appliances Co., Foshan, China). According to the mass before and after drying, apples had 17.6% of dry matter and 82.4% of water. Dry material was ground in a coffee grinder and stored under a vacuum in a plastic bag at −18 °C.

### 4.3. Extraction of Phenolic Compounds

The amounts of phenolic compounds before digestion were determined with chemical and enzyme-assisted extractions. In chemical extraction, around 0.08 g of dry apple material and 1.5 mL of 80% methanol in water were placed in a plastic cuvette, and extraction was conducted with an ultrasonic bath (RK-100, Bandelin electronic GmbH, Berlin, Germany) (15 min). The extract was centrifuged (Eppendorf Minispin, Eppendorf, Hamburg, Germany) (5 min 10,000 rpm) and separated from the residue. The residue was extracted one more time with 0.5 mL of 80% methanol with the same procedure, and the extracts were combined, filtered (0.20 μm PTFE syringe filter), and analyzed with high-performance liquid chromatography (HPLC). The extraction process assisted with enzymes was conducted on the residue that remained after chemical extraction. Distilled water (1.05 mL), bile salt (60 μL), pancreatin (30 μL), and pepsin (15 μL) were added to the residue. The solution was incubated in a dry block thermostat (Bio TDB-100, Biosan, Riga, Latvia) (37 °C, 2 h), put in an ice bath, centrifuged (5 min, 10,000 rpm), and put in an ice bath again. The extract was separated from the residue, filtered (0.20 μm PTFE syringe filter), and analyzed with the HPLC. The amounts of phenolic compounds from chemical and enzyme-assisted extractions were added, and this value represents the amount before digestion.

### 4.4. Simulated Digestion

The digestion of apples was conducted by simulating the gastric and intestinal phases without β-glucan (control) or with added β-glucan.

Gastric digestion: Around 0.08 g of dried whole apples and 175 μL of SSF, 48.8 μL of H_2_O, 1.25 μL of CaCl_2_, and 25 μL of α-amylase were placed into a plastic cuvette to simulate the salivary phase of digestion. The mixture was vortexed and put in a dry block thermostat (37 °C) for 2 min. Gastric digestion was continued by adding 375 μL of SGF, 14.8 μL of H_2_O, 0.25 μL of CaCl_2_, 10 μL of HCl (1 M), and 100 μL of pepsin into the mixture. The reaction mixture in the cuvette was put in a dry block thermostat (37 °C) for 2 h with occasional shaking in the vortex.

Intestinal digestion: The salivary and gastric phases of digestion were conducted according to the already described procedure. Intestinal digestion was continued by adding 550 μL of SIF, 180.5 μL of H_2_O, 2 μL of CaCl_2_ (0.3 M), 7.5 μL of NaOH (1 M), 250 μL of pancreatin, and 10 μL of bile salt to the solution after salivary and gastric digestion. Reaction mixture was put in a dry block thermostat (37 °C) for 2 h with occasional shaking in the vortex.

Samples obtained after gastric digestion and intestinal digestion were put in an ice bath and then in a freezing vial storage box. After cooling, the cuvettes were centrifuged (10,000 rpm, 5 min), and supernatants were filtered (0.20 μm PTFE syringe filter) and analyzed with the HPLC system.

Gastric and intestinal phases of digestion were conducted with the same described procedures but with the addition of β-glucan. B-glucan was added in two amounts, a lower one (β-glucan 1, 0.0028 g) and a higher one (β-glucan 2, 0.0041 g), which were chosen according to the dietary fibers already present in apples. Fresh apples contain around 3% of fibers [19,20]. Apples in this study contained 17.6% of dry matter and 82.4% of water. Accordingly, 3% of fibers in fresh apples make up about 17% of fibers in the dry apple material. Amounts of β-glucan were calculated according to 0.08 g of dry apple material in the simulated digestion, which contains 17% (0.0136 g) of fibers. The amounts of β-glucan added were 20% (β-glucan 1, 0.0028 g) and 30% (β-glucan 2, 0.0041 g) of the amount that could be present in the dry apple material in digestion.

The decrease in released phenolic groups with added β-glucan was calculated according to the amounts released without the addition of β-glucan.

(1) $$the\;decrease\;\left(\%\right)=\frac{{amount}_{digestion}-{amount}_{digestion\;with\;\beta -glucan}}{{amount}_{digestion}}\times 100$$

Amounts were expressed in mg kg^−1^ fw.

To calculate the adsorption capacity of β-glucan for phenolic compounds (mg per g of β-glucan), first, the amounts of phenolic groups released in digestion were recalculated in their released masses (mg). The adsorption capacity was calculated as the amount of phenolic groups adsorbed onto β-glucan by using the following equation:

(2) $$adsorption\;capacity\;\left(mg\;g^{-1}\right)=\frac{m_{digestion\;phase}-m_{digestion\;phase\;\beta -glucan}}{m_{\beta -glucan}}$$

Masses of phenolic groups released in different digestion phases (*m*_digestion phase_) and in different digestion phases with added β-glucan (*m*_digestion phase β-glucan_) were expressed in mg, and the mass of β-glucan in g.

### 4.5. Bioaccessibility Calculation

Bioaccessibility is the amount of phenolic compounds in the gastrointestinal tract released from the food matrix and accessible for absorption. It was calculated as the recovery of phenolic compounds in the stomach and small intestine:

(3) $$\left(recovery\right)\left(\%\right)=\frac{\gamma_{digestion\;phase}(mg\;{kg}^{-1})}{\gamma_{before\;digestion}(mg\;{kg}^{-1})}100$$

where γ_digestion phase_ is the amount of a phenolic compound after the gastric or intestinal digestion phase (mg kg^−1^ fresh weight (FW)), and γ_before digestion_ is the amount of a phenolic compound before digestion determined with chemical and enzyme-assisted extraction (mg kg^−1^ FW).

### 4.6. HPLC Method

By using mobile phases A (0.1% H_3_PO_4_) and B (100% methanol) and a gradient (0 min with 5% B, 5 min with 25% B, 14 min with 34% B, 25 min with 37% B, 30 min with 40% B, 34 min with 49% B, 35 min with 50% B, 58 min with 51% B, 60 min with 55% B, 62 min with 80% B, 65 min with 80% B, 67 min with 5% B, and 72 min with 5% B), phenolic compounds from samples were separated and quantified on the HPLC system (1260 Infinity II, with a quaternary pump, a PDA detector, and a vial sampler) (Agilent technology, Santa Clara, CA, USA). The HPLC system was equipped with a Poroshell 120 EC C-18 column (4.6 × 100 mm, 2.7 μm) and a Poroshell 120 EC-C18 4.6 mm guard column from the same producer. Flow rate was set to 0.5 mL min^−1^. Phenolic compounds were identified by spiking samples with authentic standards and by comparing their retention times and UV–vis spectra with those of standards. Quantification was carried out based on the calibration curves. Quercetin-3-xyloside and *p*-coumaroylquinic acid were tentatively identified with the help of reference [16] and the UV–vis spectra in that reference and quantified by using the calibration curves of quercetin-3-glucoside and *p*-coumaric acid, respectively.

### 4.7. DPPH Method

The antiradical activity was determined with the DPPH method for all samples (phenolic compounds before digestion and phenolic compounds released in the stomach and small intestine without or with added β-glucan). DPPH was prepared in methanol (1 mM). The reaction solution in a plastic cuvette contained 200 μL of DPPH reagent, a sample (10, 20, 50, 80, or 100 μL), and methanol up to total of 3000 μL. The absorbance *A*_sample_ was measured in time (2, 5, 10, and 20 min) against the blank (contains everything but the DPPH reagent) on a spectrophotometer (UV 1280, Shimadzu, Kyoto, Japan). The absorbance of the DPPH solution (2800 μL of methanol and 200 μL of DPPH) was also measured *A*_DPPH_. The inhibition of DPPH radicals was calculated.

(4) $$DPPH\;inhibition\left(\%\right)=\frac{A_{DPPH}-A_{sample}}{A_{DPPH}}*100$$

The inhibition of DPPH radicals (%) caused by all concentrations of samples after 5, 10, and 20 min was plotted against the amount of total phenolic compounds in reaction solutions (mg). Linear equations were obtained for 5, 10, and 20 min of reaction (% inhibition = a x + b, where x is mg of polyphenols). Only the linear equations for 10 min were used to calculate the value of EC_50_ (mg of polyphenols needed to inhibit 50% of DPPH radicals). Lower EC_50_ values represent higher antiradical activity. In addition, linear equations for the cases of 5, 10, and 20 min, were used to calculate the percent inhibition of DPPH radicals caused by the same amount of polyphenols (0.002 mg). In this way, the amount of DPPH radicals that can be inhibited by the same amount of phenolic compounds can be compared.

### 4.8. Statistical Analysis of the Data

All experiments of gastrointestinal digestion were performed in triplicate, and samples were analyzed once with the HPLC system (n = 3). The results are reported as mean ± standard deviation. Antiradical activity was analyzed two times. To find differences between results, the data were analyzed by using post hoc Tukey test, regression analysis, a sign test of differences, and principal component analysis (Minitab LLC., State College, PA, USA).

## 5. Conclusions

Phenolic compounds were accessible for absorption in the stomach, in a similar amount as in the native apples, and the accessible amount decreased in the small intestine. There were differences in the compositions of phenolic compounds in the stomach and small intestine. In the stomach, the major compounds were flavan-3-ols and phenolic acids, while in the small intestine, flavan-3-ols, phenolic acids, and flavonols dominated. In addition, some anthocyanins and flavan-3-ols were not identified in the small intestine or they were found in lower amounts. Phenolic acids which were not identified in the stomach were identified in the small intestine (neochlorogenic acid and cryptochlorogenic acid). These differences might need to be considered when explaining the beneficial effects of phenolic compounds from apples on the gastrointestinal tract. According to the PCA, regression analysis, and sign test, β-glucan decreased the amount of released phenolic compounds in the stomach and small intestine, probably due to the adsorption of phenolic compounds onto β-glucan. Phenolic compounds after digestion in the stomach and small intestine showed antiradical activity. The stronger inhibitors of free radicals might be the phenolic compounds in the small intestine. B-glucan lowered the antiradical activity, but not significantly.

The fact that native phenolic compounds are present in the gastrointestinal tract (stomach and small intestine) in significant amounts and show antiradical activity should be considered as an important aspect of their potential beneficial effects. The interactions with dietary fibers could be relevant for beneficial effects since those two components of the food matrix are present together in the gastrointestinal tract. Even though dietary fibers lowered the amount of released phenolic compounds and their antiradical activity, there can be important beneficial consequences that should be studied further, such as the following:

- One is the potential role of dietary fibers to serve as carriers of phenolic compounds to the lower part of the gastrointestinal tract where they can be released and act beneficially.
- Another is the potential effect of dietary fiber–phenolic compound complexes that might be created inside the stomach and small intestine.
- Furthermore, studies to enlighten whether better health effects might be achieved by consuming phenolic compounds alone, between meals, or with dietary fibers during meals, should be conducted.
- Such specific studies can be focused on phenolic compounds from apples and food sources rich in β-glucans since they are a regular part of the diet.

These studies can be used in novel areas of food science or in pharmacology for developing dietary supplements.

## Figures and Tables

**Table 1:** The amounts of phenolic compounds in apples (mg kg^−1^ fw) before digestion and in the gastric and intestinal phases of digestion without or with added β-glucan. The values are shown as the averages and standard deviations for three measurements.

	Before Digestion	Gastric Phase	Gastric Phase with β-Glucan 1	Gastric Phase with β-Glucan 2	Intestinal Phase	Intestinal Phase with β-Glucan 1	Intestinal Phase with β-Glucan 2
Anthocyanins							
cyanidin-3-galactoside	26.0 ± 2.9 ^a^	7.8 ± 1.4 ^b^	7.2 ± 1.9 ^b^	6.4 ± 1.0 ^b^	9.0 ± 5.2 ^b^	7.7 ± 0.7 ^b^	6.9 ± 4.0 ^b^
cyanidin-3-glucoside	6.1 ± 0.7 ^a^	3.6 ± 0.6 ^b^	3.5 ± 0.7 ^b^	2.9 ± 0.3 ^b^			
Total	32.1 ± 3.6 ^a^	11.4 ± 2.0 ^b^	10.7 ± 2.6 ^b^	9.3 ± 1.3 ^b^	9.0 ± 5.2 ^b^	7.7 ± 0.7 ^b^	6.9 ± 4.0 ^b^
Flavan-3-ols							
procyanidin B1	38.7 ± 2.0 ^b^	86.4 ± 6.3 ^a^	76.1 ± 18.5 ^a^	77.1 ± 14.2 ^a^			
(+)-catechin	28.0 ± 4.6 ^a^	26.3 ± 1.0 ^a^	24.2 ± 5.0 ^a^	24.0 ± 0.6 ^a^	23.8 ± 9.8 ^a^	21.1 ± 1.7 ^a^	16.6 ± 2.6 ^a^
procyanidin B2	105.9 ± 7.5 ^a,b^	152.8 ± 17.5 ^a^	146.2 ± 77.4 ^a^	127.6 ± 32.1 ^a^	24.8 ± 20.9 ^b,c^	11.9 ± 2.1 ^c^	11.4 ± 1.7 ^c^
(−)-epicatechin	194.5 ± 18.3 ^a^	80.4 ± 10.7 ^b^	73.6 ± 18.4 ^b^	67.9 ± 11.1 ^b^	55.8 ± 16.8 ^b^	54.8 ± 3.5 ^b^	48.6 ± 2.0 ^b^
Total	367.1 ± 32.4 ^a^	345.8 ± 35.5 ^a^	320.1 ± 119.4 ^a^	296.7 ± 57.9 ^a^	104.5 ± 47.4 ^b^	87.7 ± 7.3 ^b^	76.7 ± 6.3 ^b^
Dihydrochalcones							
phloretin-2-glucoside	39.5 ± 4.1 ^a^	27.0 ± 3.0 ^b,c^	25.0 ± 2.0 ^b,c^	21.5 ± 1.5 ^c^	29.1 ± 2.9 ^b^	24.3 ± 1.5 ^b,c^	21.2 ± 2.6 ^c^
Total	39.5 ± 4.1 ^a^	27.0 ± 3.0 ^b,c^	25.0 ± 2.0 ^b,c^	21.5 ± 1.5 ^c^	29.1 ± 2.9 ^b^	24.3 ± 1.5 ^b,c^	21.2 ± 2.6 ^c^
Phenolic acids							
neochlorogenic acid					8.7 ± 1.6 ^a^	9.2 ± 1.5 ^a^	7.6 ± 0.4 ^a^
chlorogenic acid	110.9 ± 6.5 ^a^	109.8 ± 3.5 ^a^	107.6 ± 7.2 ^a^	106.7 ± 9.3 ^a^	80.2 ± 8.9 ^b^	82.3 ± 4.4 ^b^	79.8 ± 4.4 ^b^
cryptochlorogenic acid					8.8 ± 1.1 ^a,b^	9.6 ± 1.2 ^a^	7.6 ± 0.6 ^b^
*p*-coumaroylquinic acid	28.4 ± 2.8 ^a^	19.5 ± 2.8 ^b^	19.3 ± 2.3 ^b^	17.3 ± 1.9 ^b^	22.7 ± 3.5 ^a,b^	21.8 ± 1.4 ^b^	20.6 ± 0.7 ^b^
total	139.3 ± 9.3 ^a^	129.2 ± 6.3 ^a^	126.9 ± 9.5 ^a^	124.0 ± 11.2 ^a^	120.3 ± 15.1 ^a^	122.9 ± 8.5 ^a^	115.6 ± 6.0 ^a^
Flavonols							
quercetin-3-galactoside	9.2 ± 1.7 ^a^	9.5 ± 2.2 ^a^	8.0 ± 3.9 ^a^	6.3 ± 0.3 ^a^	6.2 ± 1.8 ^a^	6.2 ± 0.5 ^a^	6.8 ± 1.7 ^a^
quercetin-3-rutinoside	8.4 ± 1.0 ^a^	4.5 ± 0.5 ^b^	4.2 ± 0.8 ^b^	3.6 ± 0.2 ^b^	5.0 ± 0.9 ^b^	4.8 ± 0.3 ^b^	4.5 ± 0.2 ^b^
quercetin-3-glucoside	28.8 ± 3.6 ^a^	14.6 ± 1.8 ^b^	13.7 ± 2.7 ^b^	11.6 ± 0.8 ^b^	16.6 ± 3.4 ^b^	16.0 ± 1.1 ^b^	15.0 ± 0.5 ^b^
quercetin-3-xyloside	28.6 ± 3.4 ^a^	13.5 ± 1.9 ^b^	12.8 ± 2.2 ^b^	10.8 ± 0.9 ^b^	16.3 ± 3.3 ^b^	15.8 ± 1.2 ^b^	14.7 ± 0.3 ^b^
quercetin-3-rhamnoside	9.2 ± 1.1 ^a^	7.7 ± 1.3 ^a,b^	7.0 ± 1.7 ^a,b^	5.8 ± 0.3 ^b^	7.2 ± 1.2 ^a,b^	6.9 ± 0.0 ^a,b^	6.6 ± 1.3 ^a,b^
Total	84.1 ± 10.9 ^a^	49.8 ± 7.7 ^b^	45.7 ± 11.3 ^b^	38.2 ± 2.5 ^b^	51.2 ± 10.6 ^b^	49.6 ± 3.2 ^b^	47.6 ± 4.0 ^b^
TOTAL	662.1 ± 60.2 ^a^	563.2 ± 54.5 ^a^	528.5 ± 144.6 ^a^	489.7 ± 74.3 ^a,b^	314.1 ± 81.2 ^b,c^	292.1 ± 21.2 ^c^	268.0 ± 22.9 ^c^

Different lowercase letters in the same row represent a significant difference (*p* < 0.05), respectively, obtained with the post hoc Tukey test.

**Table 2:** Percentage inhibition of DPPH radicals after 5, 10, and 20 min of reaction caused by same amounts of phenolic compounds in samples.

Sample	% Inhibition		
	5 Min	10 Min	20 Min
before digestion	14.3 ± 3.9 ^c^	18.7 ± 3.4 ^b^	24.1 ± 1.3 ^b,c,d^
gastric	12.0 ± 0.2 ^c^	14.9 ± 0.2 ^b^	20.0 ± 0.1 ^c,d^
gastric β-glucan 1	8.9 ± 1.1 ^c^	11.6 ± 1.4 ^b^	16.1 ± 1.8 ^d^
gastric β-glucan 2	8.2 ± 0.1 ^c^	10.8 ± 0.1 ^b^	14.9 ± 0.4 ^d^
intestinal	28.3 ± 7.9 ^a,b^	32.8 ± 7.2 ^a^	36.3 ± 6.9 ^a^
intestinal β-glucan 1	30.4 ± 2.1 ^a^	33.3 ± 2.1 ^a^	32.6 ± 1.7 ^a,b^
intestinal β-glucan 2	15.4 ± 0.0 ^bc^	19.4 ± 1.4 ^b^	30.4 ± 1.4 ^a,b,c^

% inhibition calculated for the same amount of phenolic compounds in the sample (0.002 mg of total phenolic compounds). Different letters in the same column represent statistically different values, respectively, according to the post hoc Tukey test.

## Data Availability

The original contributions presented in this study are included in the article/Supplementary Material; further inquiries can be directed to the corresponding author.

## Supplementary Materials

The following supporting information can be downloaded at https://www.mdpi.com/article/10.3390/molecules30020301/s1, Figure S1: The distribution of phenolic groups before digestion and in gastric and intestinal digestion expressed in percentages of the total released amount; Figure S2: Examples of the regression analysis of the total released phenolic compounds or flavan-3-ols vs. different phases of digestion (all digestion phases with or without added β-glucan); Figure S3: The percentage of inhibition of DPPH radicals by phenolic compounds from apples (before digestion) and by samples after digestion in the stomach and small intestine in relation to the time (samples were added in different volumes (10, 20, 50, 80, and 100 μL) in reaction mixtures); Table S1: The results of the regression analysis of the amounts of released phenolic compounds vs. all different digestion phases (the slope of the regression line, its standard error, and the T and P values); Table S2: Linear regression of phenolic compound amount, Y, versus digestion phase and β-glucan amount; Table S3: Drops in mean phenolic compound amounts from adding β-glucan (for sign test); Table S4: Antiradical activity before digestion and in the gastric and intestinal phases of digestion without or with added β-glucan, expressed as the EC_50_ value after 10 min of reaction.
