# Supplementary material for: The Effect of β-Glucan on the Release and Antiradical Activity of Phenolic Compounds from Apples in Simulated Digestion
Source: Molecules. 2025 Jan 14;30(2):301. doi: 10.3390/molecules30020301 (PMC11768063; doi:10.3390/molecules30020301)
Supplement: Supplementary file 1 [file molecules-30-00301-s001.zip › molecules-3350429-supplementary.pdf]

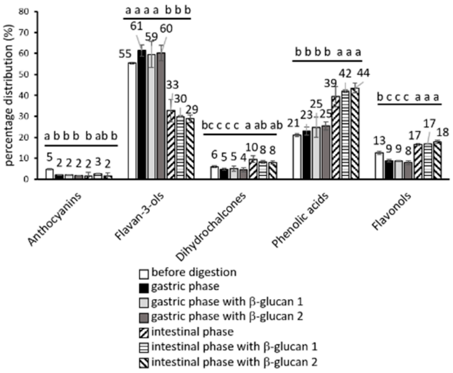

Figure S1. The distribution of phenolic groups before digestion and in gastric and intestinal digestion expressed in percentages of the total released amount

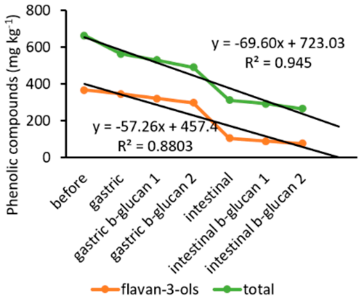

Figure S2. Examples of the regression analysis of the total released phenolic compounds or flavan-3-ols *vs.* different phases of digestion (all digestion phases with or without added β-glucan).

Table S1. The results of the regression analysis of the amounts of released phenolic compounds *vs.* all different digestion phases (the slope of the regression line, its standard error, and T and P values)

Phenolic compounds	Slope	Standard error	T value	P value
**Phenolic compounds *vs* all phases of digestion without or with β-glucan ***				
Anthocyanins	-3.388	0.683	-4.96	0.000
Flavan-3-ols	-57.26	7.06	-8.11	0.000
Dihydrochalcones	-2.002	0.540	-3.71	0.001
Phenolic acids	-3,225	0.930	-3.47	0.003
Flavonols	-3.72	1.49	-2.51	0.021
Total	-69.60	7.61	-9.15	0.000

*All phases of digestion - before digestion, gastric digestion, gastric digestion with different amounts of β-glucan, intestinal digestion, intestinal digestion with different amounts of added β-glucan

Table S2. Linear regression of phenolic compound amount, Y, versus digestion phase and β-glucan amount

Y	Intercept	X_1 = phase (1= gastric, 2 = intestinal)				X_2 = β-glucan (0 = none, 1= low, 2 = high)			
**Amounts of Polyphenols**		**Coefficient**	**SE**	**T**	**P**	**Coefficient**	**SE**	**T**	**P**
Anthocyanins	14.20	-2.66	0.72	-3.71	0.003	-1.06	0.45	-2.35	**0.035**
Flavan-3-ols	571.40	-231.30	24.50	-9.43	0.000	**-19.20**	15.00	-1.28	0.220
Dihydrochalcones	27.49	0.37	1.03	0.36	0.724	-3.35	0.63	-5.31	**0.000**
Phenolic acids	136.30	-7.11	4.08	-1.74	0.102	-24.70	2.50	-0.99	0.338
Flavonols	43.45	4.91	3.07	1.60	0.131	-3.79	1.88	-2.01	0.062
Total	794.40	-237.50	30.60	-7.75	0.000	**-29.70**	18.80	-1.59	0.134

Table S3. Drops in mean phenolic compound amounts from adding β-glucan (for sign test)

Digestion phase	Anthocyanins mg kg^{-1} fw	Flavan-3-ols mg kg^{-1} fw	Dihydrochalcones mg kg^{-1} fw	Phenolic acids mg kg^{-1} fw	Flavonols mg kg^{-1} fw
gastric minus gastric β-glucan1	-0.71	-25.7	-1.98	-2.33	-4.01
gastric β-glucan 1 minus gastric β-glucan 2	-1.41	-23.4	-3.55	-2.89	-7.55
Intestinal minus intestinal β-glucan 1	-1.34	-16.7	-4.86	+2.54	-1.63
intestinal β-glucan 1 minus intestinal β-glucan 2	-0.76	-11.1	-3.03	-7.27	-1.96

Binomial probability of 1 or fewer increases in 20 observations = P = 0.00002 (reject null hypothesis of no β-glucan effect)

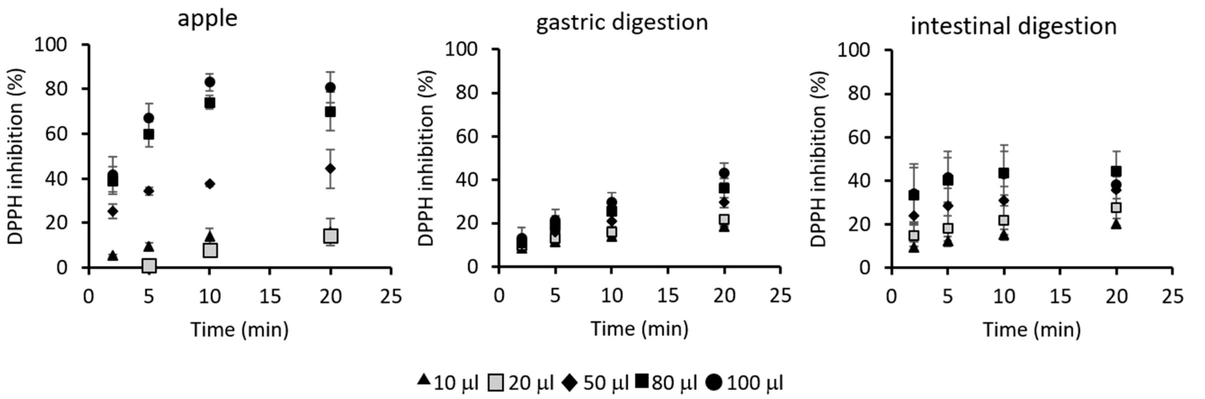

Figure S3. The percentage of inhibition of DPPH radicals by phenolic compounds from apples (before digestion) and by samples after digestion in the stomach and small intestine in relation to the time (samples were added in different volumes (10, 20, 50, 80, and 100 µL) in reaction mixtures).

Table S4. Antiradical activity before digestion and in the gastric and intestinal phases of digestion without or with added β-glucan, expressed as the EC_{50} value after 10 min of reaction

Digestion phases	EC_{50} (µg)
before digestion	5.3 ± 0.2[b]
gastric	28.8 ± 8.0[*a]
gastric β-glucan 1	25.0 ± 1.3[*a]
gastric β-glucan 2	25.8 ± 1.6[*a]
intestinal	3.7 ± 0.8[b]
intestinal β-glucan 1	3.3 ± 0.3[b]
intestinal β-glucan 2	5.0 ± 0.0[b]

Antiradical activity determined with DPPH method, expressed as EC_{50} value after 10 min of reaction. EC_{50} value represents the amount of total phenolic compounds present in the sample needed to inhibit 50 % of DPPH radical. Lower EC_{50} values represent stronger antiradical activity.

* EC_{50} value calculated before samples reached a steady state.

Different lower case letters in the column represent a significant difference (p<0.05) obtained with the post-hoc Tukey test
